# Interleukins as Mediators of the Tumor Cell—Bone Cell Crosstalk during the Initiation of Breast Cancer Bone Metastasis

**DOI:** 10.3390/ijms22062898

**Published:** 2021-03-12

**Authors:** Marie-Therese Haider, Nicole Ridlmaier, Daniel J. Smit, Hanna Taipaleenmäki

**Affiliations:** 1 Molecular Skeletal Biology Laboratory, Department of Trauma and Orthopedic Surgery, University Medical Center Hamburg-Eppendorf, 20246 Hamburg, Germany; m.haider@uke.de (M.-T.H.); nicole.ridlmaier@gmx.at (N.R.); 2 Department of Life Sciences, IMC FH Krems University of Applied Sciences, 3500 Krems, Austria; 3 Institute of Biochemistry and Signal Transduction, University Medical Center Hamburg-Eppendorf, 20246 Hamburg, Germany; d.smit@uke.de

**Keywords:** breast cancer, bone metastasis, interleukin, disseminated tumor cell, bone microenvironment, dormancy

## Abstract

Patients with advanced breast cancer are at high risk of developing bone metastasis. Despite treatment advances for primary breast cancer, metastatic bone disease remains incurable with a low relative survival. Hence, new therapeutic approaches are required to improve survival and treatment outcome for these patients. Bone is among the most frequent sites of metastasis in breast cancer. Once in the bone, disseminated tumor cells can acquire a dormant state and remain quiescent until they resume growth, resulting in overt metastasis. At this stage the disease is characterized by excessive, osteoclast-mediated osteolysis. Cells of the bone microenvironment including osteoclasts, osteoblasts and endothelial cells contribute to the initiation and progression of breast cancer bone metastasis. Direct cell-to-cell contact as well as soluble factors regulate the crosstalk between disseminated breast cancer cells and bone cells. In this complex signaling network interleukins (ILs) have been identified as key regulators since both, cancer cells and bone cells secrete ILs and express corresponding receptors. ILs regulate differentiation and function of bone cells, with several ILs being reported to act pro-osteoclastogenic. Consistently, the expression level of ILs (e.g., in serum) has been associated with poor prognosis in breast cancer. In this review we discuss the role of the most extensively investigated ILs during the establishment of breast cancer bone metastasis and highlight their potential as therapeutic targets in preventing metastatic outgrowth in bone.

## 1. Introduction

### 1.1. Breast Cancer Bone Metastasis

Although breast cancer remains the most frequently diagnosed cancer in women worldwide, survival rates for patients with primary breast cancer have steadily improved [1]. However, metastatic disease often establishes several years after successful treatment of the primary tumor. Unfortunately, the 5-year relative survival rates for patients with distant, metastatic breast cancer remain very low, with less than 30 percent of patients surviving their disease for longer than 5 years [1,2]. Besides lung, liver and brain, breast cancer metastases primarily manifest in bone, where the disease is accompanied by excessive, osteoclast-mediated bone destruction. At this stage treatment remains palliative and patients suffer from associated skeletal related events (SREs) including fractures and pain. Upon proliferation in bone, tumor cells perturb the tightly balanced bone remodeling cycle through secretion of factors such as parathyroid hormone-related protein (PTHrP) and interleukins (ILs) that stimulate osteoblasts to produce increased levels of receptor activator of NF-κB ligand (RANKL). RANKL binds to its cognate receptor RANK on osteoclasts and stimulates osteoclast precursors to differentiate into mature, bone resorbing osteoclasts. The increased osteoclastogenesis and enhanced bone resorption results in the release of tumor growth promoting factors (e.g., transforming growth factor-β (TGF-β)) from the resorbed bone matrix [3,4]. Thus, the vicious cycle of bone metastasis is initiated, highlighting the importance of the bone cell-tumor cell crosstalk in disease progression.

Given that the disease cannot be cured once it is progressing in bone, treatment approaches that target the early stages of disease establishment such as tumor cell colonization and escape from dormancy, would be needed to provide better prognosis and treatment outcome for patients with breast cancer bone metastasis. Upon their arrival in bone, disseminated tumor cells (DTCs) encounter a heterogeneous microenvironment that is comprised of cellular and molecular entities that vastly differ from the original environment of the primary tumor. Already in 1889, Steven Paget proposed in his “seed and soil” theory that DTCs (“seeds”) have to encounter a fruitful environment (“soil”) that is suitable for their growth at secondary sites [5,6]. Paget proposed that bone provides such a soil for DTCs due to the constant availability of growth supporting factors that are released during bone remodeling. Over the last years research has improved our understanding of the metastatic process and specialized environments that control tumor cell dormancy and metastatic outgrowth at secondary sites are predominantly referred to as “metastatic niches”. In bone, the metastatic niche is comprised of a vascular- (vascular endothelial cells), hematopoietic stem cell-, and endosteal niche (osteoblasts, osteoclasts, fibroblasts) [7,8]. Additionally, several other cell types including adipocytes, osteocytes, immune cells and megakaryocytes are increasingly demonstrated to mediate metastatic growth in bone [8,9,10,11,12]. It remains impossible to completely separate these niches and recent studies also suggest that tumor cells preferentially colonize areas where they overlap [13]. Besides direct cell-cell contact, soluble factors mediate cellular communication in the metastatic niche. In this context, interleukins (ILs), cytokines with immunomodulatory functions, are considered as key regulators of the bone cell-DTC crosstalk. However, current literature does not provide a complete overview of the role of ILs during the initial stages of breast cancer bone metastasis including the recruitment of circulating tumor cells to the metastatic site, maintenance and escape form dormancy as well as colonization of the metastatic site. Here, we review and discuss the role of the best-validated ILs (IL-1β, IL-6, IL-8 and IL-11) involved in the bone cell—breast cancer cell crosstalk during the early events of breast cancer bone metastasis.

### 1.2. Interleukins

Interleukins were discovered as small secreted signaling proteins that mediate communication between immune cells, in particular leukocyte communication (reviewed in great detail in [14]). Indeed, the majority of ILs are produced by immune cells [15]. However, several other cell types including bone cells and tumor cells are now known to produce ILs and to respond to both, autocrine and paracrine IL-signaling [16,17,18,19,20]. By binding to their cognate cell surface receptor, ILs modify several physiological cell processes including proliferation, differentiation and apoptosis. Since the discovery of IL-1 in 1977, in total 38 ILs have been identified with distinct signaling and biological properties (Table 1). Several classification systems exist but briefly ILs are grouped into families according to biological function, sequence homology and/or receptor chain similarities [21,22]. With regards to the receptors this includes for example type I and type II cytokine receptor families as well as the interleukin-1 receptor/Toll-like receptor (IL-1R/TLR) [23]. Signaling via type I and type II receptors including for example IL-6 or IL-11 involves intracellular signaling cascades such as mitogen-activated protein kinase (MAPK), extracellular-signal-regulated kinase (ERK), Janus kinase/signal transducer and activator of transcription (JAK/STAT), and the phosphoinositide 3-kinase (PI3-K) [23]. Signaling downstream the IL-1R/TLR axis involves TNF receptor associated factor 6 (TRAF6), nuclear factor-kappa B (NFkB), c-Jun N-terminal kinase (JNK) and p38 [23]. Of note, whereas IL-1, IL-6 and IL-11 belong to the interleukin family of cytokines, IL-8 belongs to the CXC-motive chemokine family [24]. It is therefore also referred to as CXCL-8 and signals via the chemokine receptors CXCR1 and CXCR2 (Table 1) [24]. Although IL-8 is strictly speaking rather a chemokine than an interleukin, we included it in this review due to its important contribution to the establishment and progression of breast cancer bone metastasis.

### 1.3. Role of Interleukins in Physiological Bone Remodeling

Skeletal integrity is maintained through the balanced activity of osteoclast-mediated bone resorption and subsequent formation of new bone by osteoblasts [25]. Key signaling pathways that regulate bone remodeling include the RANK/RANKL/osteoprotegerin (OPG)—axis and canonical Wnt signaling. Several cytokines and growth factors as well as hormones, including parathyroid hormone (PTH), vitamin D and growth hormones act as paracrine and endocrine regulators of bone remodeling [26]. ILs have been reported to affect the function and maturation of both, osteoblasts and osteoclasts [27,28,29]. Both cell types secrete ILs and express corresponding receptors [18,30,31,32,33]. Hence, bone remodeling is partially regulated by IL-mediated crosstalk between osteoblasts and osteoclasts and includes both, autocrine and paracrine signaling.

The majority of ILs is known to act pro-osteoclastogenic, either via direct or indirect effects on osteoclast maturation, differentiation and function [34,35]. Bone marrow stromal cells produce ILs (e.g., IL-6, IL-11) which induce osteoclastogenesis in vitro [28]. IL-6 for example, can induce osteolysis via crosstalk with the prostaglandin E_2_ (PGE_2_)/Cyclooxygenase (COX-2) signaling cascade [36], which shifts the RANK/RANKL/OPG ratio towards enhanced osteoclastogenesis [36]. However, ILs can also induce osteoclastogenesis independent of RANKL through JAK1/STAT3 signaling [37].Whereas the published effects of ILs on osteoclasts predominantly report a pro-osteoclastic role, the effects on osteoblasts are varied, depending on the interleukin as well as on the study models employed (human vs. mouse osteoblasts, time and dose of stimulation) [17,18,33,36,38,39]. For instance, depending on the time of stimulation, IL-1β has been reported to have biphasic effects on osteoblast differentiation in vitro [38].

Importantly, in response to factors released during bone resorption (e.g., TGF-β), bone marrow stromal cells, including osteoblasts and fibroblasts, are thought to produce elevated levels of ILs, that could enhance bone resorption [40,41] and thus a feed-forward loop. Indeed, upregulation of ILs has been implicated in several skeletal disorders including rheumatoid arthritis, osteoporosis or cancer-induced bone disease [33,42,43,44].

### 1.4. ILs and Breast Cancer Bone Metastasis

Breast cancer bone metastases are predominantly osteolytic, caused by excessive, osteoclast-mediated bone destruction [45]. Given the osteoclast-promoting role of ILs in physiological bone remodeling, their role in breast cancer bone metastasis is evident. Indeed, serum levels of IL-11 [46] and IL-8 [47] are reported to be elevated in patients with breast cancer bone metastases when compared to patients with primary breast cancer. Additionally, patients with multiple sites of breast cancer metastases had higher serum IL-6 levels compared to patients with one documented metastatic site [48,49]. Additionally, patients with high levels of IL-6 had a significantly reduced survival compared to patients with lower IL-6 [48]. Furthermore, analysis of tissue samples from breast cancer patients (stage II/III) revealed that the expression of IL-1β in tumor cells correlated with breast cancer recurrence in bone [50]. Moreover, the expression of IL-1β, IL-6, IL-8 or IL-11 in breast cancer cells has been associated with their metastatic potential and aggressive behavior [20,37,51,52,53]. Additionally, elevated levels of IL-1β and IL-6 have been detected in serum of mice bearing metastatic tumors [9]. Pre-clinical studies have recently highlighted the importance for targeting IL-signaling early on in metastatic breast cancer [54,55]. Here preventative administration of agents that inhibit IL-signaling was more effective in reducing and/or preventing breast cancer bone metastasis and osteolytic bone disease in vivo than treatment schedules starting after tumor cell colonization in bone [54,55]. However, in the clinical setting many challenges remain regarding preventative treatment. First of all, the disease is commonly diagnosed at later stages, when the tumor is already progressing in bone, whereas it remains impossible to detect single, disseminated, dormant tumor cells in bone. Another challenge is to establish which patient population would benefit from preventative treatment as well as the time point at which preventative treatment would be administered. Nevertheless, these approaches could be useful to establish novel treatment approaches for patients with metastatic breast cancer.

## 2. The Role of ILs during the Establishment and Progression of Bone Metastasis

### 2.1. Cancer Stem Cells, Circulating Tumor Cells and IL-Mediated Attraction to the Bone Metastatic Niche

Only a small subset of cancer cells is thought to be able to initiate metastatic growth. Emerging evidence has demonstrated that within tumors a subpopulation of cancer cells with stem cell-like features exists. This subpopulation is referred to as cancer stem cells (CSCs) and has the ability to self-renew, differentiate and initiate metastatic outgrowth (colonization) [56]. In addition, CSCs are also critical in regulating resistance to anti-cancer therapy [57]. Recently, bone marrow-derived factors, including ILs, have been suggested to specifically enhance breast CSCs to form colonies upon their arrival at the metastatic site [58].

Indeed, medium conditioned by human bone marrow cultures stimulated CSC colony formation and mammosphere self-renewal of several breast cancer cell lines or samples obtained from early breast cancer patients (estrogen receptor positive and negative (ER^+^ and ER^−^)) [58]. Furthermore, aldehyde dehydrogenase (ALDH), a marker for CSCs, was reduced in MCF-7 cells upon bone conditioned medium stimulation in these studies. Moreover, breast cancer cells that were isolated from mouse bones had significantly higher formation of mammosphere colonies when compared to cells that were grown in in vitro cultures or subcutaneously in vivo [58]. Elevated IL-levels (IL-6, IL-8, IL-15 and IL-1ß) in bone-conditioned medium could account for the increased CSC colony formation. IL-induced CSC colony formation was mediated via the IL-1ß-NFKB/CREB-Wnt signaling pathway and only neutralization of IL-1ß could revert the induction of colony formation [58]. Consequently, the authors proposed that targeting CSC via IL-1ß-Wnt signaling axis could have potential for adjuvant treatment to prevent the establishment of breast cancer bone metastases [58]. Consistently, others have recently proposed that CSCs undergo dormancy to escape from anti-estrogen treatment and that targeting the ALDH^+^ IL-1 receptor^+^ cell population could be useful to prevent treatment resistance in ER^+^ breast cancer [59].

Another prominent IL involved in breast cancer stemness includes IL-6, probably the most studied one, and several studies report a link between IL-6 signaling and CSCs [60,61,62]. For instance, signaling via the IL-6/STAT-3, Notch and EGFR signaling axis through the cell surface receptor syndecan-1 (CD138) has been established as a novel regulatory pathway responsible for the CSC phenotype in inflammatory breast cancer [60]. Syndecan-1 can act as a co-receptor for several growth factors, cytokines and chemokines and thereby regulate physiological and pathological cell processes including proliferation, migration, adhesion and differentiation [63]. Interestingly, ablation of syndecan-1 by siRNA reduced the capacity of SUM-149 cells to form colonies, a common marker for self-renewal of CSCs [60]. Of note, downregulation of syndecan-1 reduced mRNA expression of IL-6, and IL-8 in SUM-149 and SKBR3 cells. Cytokine array analysis of conditioned medium from cells transfected with syndecan-1 siRNA revealed a reduced presence of IL-6 and IL-8 upon downregulation of syndecan-1 [60]. Some years ago, others have also postulated a role of syndecan-1 in the β-integrin-, and IL-6 dependent adhesion and migration of breast cancer cells [64]. In line with these data, IL-6 has been reported to induce stemness features in breast cancer cells [62]. CD44 is commonly used to identify cancer stem cells and exposure to IL-6 (50 ng/mL for 10 days) enriched the CD44^+^ cell population in the human T47D breast cancer cell line. This cell population was also more tumorigenic when injected into the mammary fat pad of NOD/SCID mice [62]. Further studies have also suggested the potential of IL-6/CSC-targeted therapies in patients who developed resistance to hormone therapy in luminal breast cancer [61].

A correlation between IL-8 levels and mammosphere-forming efficiency of patient derived breast cancers has been reported, highlighting the role of IL-8 in regulating breast CSC activity [65]. ALDH^+^ CSCs have previously been shown to overexpress CXCR1 (IL-8 receptor) and to expand upon IL-8 stimulation [66,67]. Indeed, IL-8 has been detected in metastatic fluids of breast cancer patients, which correlated with mammosphere formation activity [65]. A small molecule antagonist of CXCR1/2 (SCH563705) in combination with lapatinib (HER2/neu and epidermal growth factor receptor inhibitor) was more effective in reducing mammosphere formation efficiency in HER2^+^ cancers when compared to single agents [65]. These data propose benefits for patents with HER2^+^ breast cancer by combining CXCR1/2 inhibitors with current HER2-targeted therapies to target disseminated CSCs [65]. In agreement, Ginestier et al. have shown that IL-8 could be used to target CSCs in breast cancer metastasis using either an antibody against the IL-8 receptor CXCR1 or repertaxin, a small molecule CXCR1 inhibitor [68]. In these studies treatment of SUM159 breast cancer cells with 100 nM repertaxin for 3 days reduced the ALDH^+^ CSC population in vitro, with similar effects being observed in vivo [68]. Furthermore, treatment with repertaxin 24 h (2× daily for 28 days) after intra cardiac injection of HCC194, MDA-MB-453 and SUM159 breast cancer cells showed a reduced development of metastases for HCC1953 and SUM159 cells. Interestingly, no effect on MDA-MB-453 cells was observed, which was explained by resistance to repertaxin due to the presence of a phosphatase and tensin homolog (PTEN) mutation [68]. Using a humanized mouse model with subcutaneously implanted bone chips in NOD/SCID mice, others demonstrated that the bone microenvironment changes the phenotype of CD44^+^CD24^−^ breast CSCs to CD44^−^CD24^+^ CSCs with elevated gene expression of IL-6 and IL-8 when they metastasized to bone [69]. Together these studies emphasize the role of ILs during the early phases of breast cancer bone metastasis and highlight the possibility of combining standard of care treatments with agents that target IL-signaling to prevent breast cancer bone metastasis.

**Figure 1:**
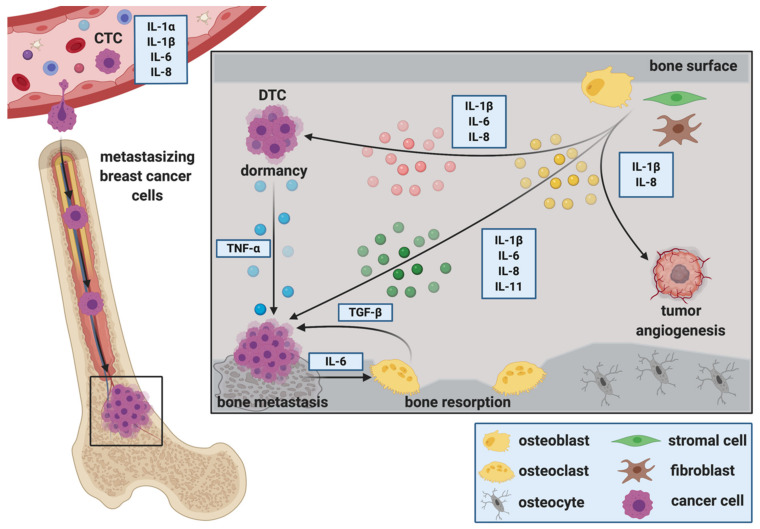
Interleukins during the progression of breast cancer bone metastasis. Interleukins (ILs) play pivotal roles in mediating several steps of the metastatic cascade in breast cancer. Dynamic changes in circulating tumor cell (CTC) levels in combination with corresponding alterations in serum IL levels might serve as prognostic markers for the progression of breast cancer. Furthermore, ILs are suggested to regulate the attraction of disseminated tumor cells (DTCs) to the metastatic site, facilitate extravasation from the circulation, adhesion and migration in the secondary organ. Once in the secondary organ, DTCs receive interleukin signals from adjacent, tissue-resident cells that regulate their fate in the secondary organ. Cells of the bone microenvironment secrete ILs that control tumor cell dormancy. In turn, proliferating tumor cells secrete ILs that stimulate osteoclastic bone resorption and consequently osteolysis. Factors released during osteolysis (e.g., Transforming growth factor β (TGF-β)) then fuel the vicious cycle of bone metastasis. Stromal-derived ILs can also regulate tumor growth via affecting vascular endothelial cells, thus promoting vascularization and metastatic growth.

Circulating tumor cells (CTCs) are often found in the blood stream of patients with solid tumors. These cells are shed into the circulation and are thought to function as seeds for metastases. Similar characteristics of CTCs compared to CSC including a high plasticity and the capability to form distant metastases have been reported previously [70]. Recently, Koch et al. established a CTC line (CTC-ITB-01) from the blood of a patient with advanced ER^+^ breast cancer [71]. CTC-ITB-01 cells were capable of forming metastases at different organs including the bone in an in vivo xenograft mouse model after intraductal injection. Further characterization of common stemness markers in breast cancer revealed a high expression of ALDH1, a more recent breast cancer stemness marker [72] in CTC-ITB-01 cells. CTCs are often associated with poor prognosis and used as clinical biomarkers or as novel therapeutic targets in oncology [73,74,75]. Dynamic changes in CTC levels in combination with corresponding alterations in serum IL levels might serve as prognostic markers for the progression of breast cancer (Figure 1) [50,76,77,78]. A study investigated if certain serum cytokine profiles (e.g., ILs, tumor necrosis factor alpha, interferon gamma) could be associated with the presence of CTCs in breast cancer patients [77]. Interestingly, in the CTC-positive group, elevated levels of serum IL-1α were observed in patients without lymph metastases when compared to patients with lymph involvement, suggesting that IL-1α is associated with the release of CTCs into the circulation rather than into the lymphatic system [77]. In mouse models of breast cancer bone metastasis elevated expression of IL-1β has been determined in CTCs when compared to breast cancer cells isolated from mammary tumors [50]. Others have associated CTC counts in patients suffering from progressing metastatic breast cancer with elevated IL-6 and IL-8 levels [76]. Others report elevated serum levels of IL-8 and IL-13 in patients with no CTCs and progesterone receptor-negative breast cancer when compared to patients with progesterone receptor-positive breast cancer [78]. In contrast, no differences in IL-levels were observed in breast cancer patients with CTCs [78]. Separate studies have found a reverse correlation of CTCs and IL-2 levels in patients with advanced breast cancer [79]. Together, these studies underscore the challenge of establishing a correlation between the levels of ILs and CTCs as prognostic markers, in particular as it might depend on the cancer subtype (e.g., receptor status).

Elevated levels of ILs in the bone microenvironment could also regulate the recruitment of CTCs to the bone as well as the formation of pre-metastatic niches. Studies by Kim and colleagues also shown that tumor-derived IL-6 and IL-8 could be involved in mediating CTCs to re-infiltrate their original tumor, a process referred to as tumor-self seeding [80]. Indeed, this model could also be translated into the setting of breast cancer bone metastasis, where increased levels of IL-6 and IL-8 in the bone environment could act as chemoattractants for CTCs.

### 2.2. The Role of ILs during the Extravasation of DTCs at the Metastatic Site

The extravasation of DTCs from the circulation into distant organs requires interaction of DTCs with endothelial and stromal cells via adhesion molecules. Interestingly, IL-1ß and IL-6 have been shown to increase focal adhesion kinase (FAK) in breast cancer cells [64,81]. Further in vitro studies have shown that osteoblasts can stimulate the adhesion of breast cancer cells to a monolayer of bone marrow endothelial cells [82]. In these experiments osteoblasts produced increased levels of inflammatory cytokines, including IL-1ß, which increased the expression of adhesion molecules on endothelial cells and consequently facilitated breast cancer cell adhesion [82]. Similarly, macrophage derived IL-1ß has been shown to support breast cancer cell adhesion to endothelial cell monolayers [83]. Also adipocytes have been reported to alter adhesion molecules in breast cancer cells via IL-8 [84]. Stimulation with recombinant human IL-8 increased mucin-1 in MCF-7 and T47D breast cancer cells and decreased intercellular adhesion molecule 1 (ICAM-1) as well as vascular cell adhesion molecule 1 (VCAM-1) in T47D cells [84]. Bendre and colleagues compared characteristics of highly metastatic MDA-MET breast cancer cells against non-metastatic MDA-231 cells in vitro [53]. Although these studies did not report differences in proliferation between the two cell lines, an increased early adhesion to type IV collagen matrix and increased invasion through matrigel was observed [53]. Microarray and gene expression analysis revealed that only a limited number of genes was differentially expressed between the two cell lines, with one of them being IL-8 [53]. Together, these studies highlight the importance of ILs in breast cancer cell adhesion, a key process in cancer progression and metastasis.

### 2.3. ILs during Migration, Invasion and Epithelial to Mesenchymal Transition (EMT) at the Metastatic Site

ILs are also implicated in the migration of tumor cells to the site of metastatic growth. Both, stromal cells as well as tumor cells can be the source of ILs, resulting in autocrine as well as paracrine signaling between bone and cancer cells. For example, overexpression of IL-1β in tumor cells has been shown to increase their migration as well as their invasion through Matrigel towards osteoblasts [50]. Others have reported that human bone tissue—conditioned medium increased the migration of MDA-MB-231-fluc-EGFP breast cancer cells when compared to control medium, which was associated with increased levels of adipokines and cytokines including IL-1ß [12]. Additionally, IL-1β enhances tumor cell invasion through supporting the production of matrix-degrading enzymes (e.g., matrix metalloprotease 9 (MMP-9)) as well as through activation of the Src/FAK pathway [81]. Similarly, osteoblast-derived cytokines including IL-6 and IL-8 have been reported to stimulate the migration of breast cancer cells in vitro [85].

Isolated fibroblasts from common sites of breast cancer metastasis, including lung and bone, produce elevated levels of IL-6 when compared to normal skin fibroblasts [86]. Fibroblast-derived IL-6 stimulated growth and invasion of breast cancer cells in vitro through activation of STAT-3 signaling [86]. Oncostatin M (OSM), an IL-6 family cytokine that is produced by tumor stromal cells such as neutrophils [87], increased breast cancer cell invasion in vitro [87,88]. IL-6 expressing breast cancer cells have also been shown to recruit myeloid-derived suppressor cells to infiltrate sites of primary as well as distant, metastatic tumor growth [89]. Metastasizing cancer cells stimulated myeloid-derived suppressor cells to secrete elevated levels of IL-6 and IL-6 receptor-α, which in turn stimulated breast cancer cell aggressiveness and metastasis [89]. Thus, an IL-6 signaling loop between breast cancer cells and myeloid derived suppressor cells that drives cancer cell invasiveness and distant metastasis has been proposed [89].

Epithelial to mesenchymal transition (EMT) is well established to be involved in breast cancer cell invasion and metastasis [90]. Vimentin, smooth-muscle actin, N-cadherin and cadherin-11 are commonly referred as markers for EMT [91]. CD44^+^ T47D breast cancer stem cells induced by IL-6 have been shown to undergo EMT in vitro, which was characterized by an increased presence of vimentin [62]. Others have shown that several breast cancer cell lines over expressing IL-1ß demonstrated elevated EMT due to higher expression of N-cadherin and decreased levels of E-cadherin [50]. Additionally, the IL-8/IL-8R signaling axis is induced in Brachyury-overexpressing breast cancer cells during EMT, which further promotes tumor progression by altering the tumor microenvironment and adjacent tumor cells [92].

### 2.4. ILs as Regulators of DTC Dormancy at the Metastatic Site

Once in the secondary organ, DTCs receive signals from adjacent, tissue-resident cells that regulate their fate in the distant site. It has been suggested that inflammatory cytokines associated with pathological bone remodeling (e.g., IL-1β and tumor necrosis factor-α (TNF-α)) may trigger escape from dormancy and the occurrence of bone metastases [93]. Others propose that stromal-derived IL-6, IL-8 and TGF-β1 stimulate breast cancer cell escape from dormancy in vitro [94] (Figure 1). However, precise mechanisms regulating tumor cell dormancy are poorly understood [95]. As breast cancer bone metastases remains incurable once progressing in bone, preventing dormant DTCs from reawakening could be a potential key to avoid cancer recurrence at secondary sites.

Bone marrow stromal cells, including osteoblasts, interact with DTCs to regulate tumor cell dormancy. Interestingly, some reports shown that osteoblasts promote breast cancer cell dormancy in bone, whereas others suggest that they support metastatic growth [93,96,97,98,99]. Recently, a special subpopulation of osteoblasts that supports breast cancer cell dormancy has been reported [100]. This subpopulation showed a reduced expression of IL-6 and α-smooth muscle actin (α-SMA) [100]. On the other hand, osteoblasts have been shown to express increased levels of inflammatory cytokines including IL-6 and IL-8 upon the presence of breast cancer cells, which facilitates metastatic breast cancer growth in bone [98,101]. Additionally, co-culture of otherwise weakly metastatic (dormant) breast cancer cells with MC3T3-E1 osteoblasts increased breast cancer growth in vitro when stimulated with IL-1ß and TNF-α [93]. Using ex-vivo co-culture systems of breast cancer cells and mouse bones, others have also shown that a CXCL5/CXCR2 (the IL-8 receptor-β) signaling axis might be involved in regulating the balance between cancer cell quiescence/dormancy and subsequent breast cancer outgrowth in bone [102]. Additionally, breast cancer cells that remain dormant within the bone are suggested to be sensitive to the leukemia inhibitory factor (LIF), which belongs to the IL-6 family of cytokines [103]. LIF binds to LIF receptor (LIFR), which has been shown to be downregulated in patients with breast cancer bone metastasis [103].Of note, several key bone cells including osteoblasts, osteoclasts, chondrocytes and adipocytes secrete LIF and also express corresponding receptors [104]. Mechanistically, loss of LIF/STAT3 signaling was associated with re-awakening of cells from dormancy. Indeed, loss of LIFR down-regulated dormancy-related genes (e.g., thrombospondin-1 (TSP1), tropomyosin-1 (TPM1), TGF-ß2) and resulted in tumor cell dissemination to bone and osteolytic disease in vivo [103]. In the liver, another common site of breast cancer metastasis, activated hepatic stellate cells have been proposed to aid MDA-MB-231 breast cancer cell escape from dormancy via elevated secretion of IL-8 [105]. In summary, IL-signaling between bone cells and DTCs could be used as a therapeutic target to prevent DTC escape from dormancy (Figure 1).

### 2.5. ILs and Breast Cancer Colonization in Bone, Metastatic Outgrowth and the Creation of a Metastasis—Supporting Environment

Upon their presence in bone, DTCs alter the microenvironment to provide suitable growth conditions. Mesenchymal derived cells including osteoblasts and fibroblasts make up a large proportion of the cellular entities that DTCs encounter upon their arrival in bone. Stimulated by DTCs, osteoblasts are suggested to produce soluble factors with chemo attractive and growth regulating functions for both, tumor cells and osteoclasts [96,98]. Thereby, they are thought to regulate the initiation and activation of the vicious cycle of bone metastasis and consequently metastatic bone destruction. Metastatic colonization is defined as the ability of cancer cells to “form a clinically relevant metastasis at a secondary cancer site(s)” [106]. DTCs have to overcome many obstacles to successfully colonize distant organs. This includes the infiltration of the distant site, evading immune attack, adapting to the new environment and surviving to eventually initiate metastasis.

#### 2.5.1. IL-1ß

In vivo, inhibition of IL-1β signaling with either a mouse anti-IL-1ß -antibody (2 mg/kg) or the human anti-IL-1β antibody anakinra (1 mg/kg/day) reduced breast cancer bone colonization in hind limbs of mice [58]. Interestingly, tumor cells were present in the bones of mice that had received anakinra but had not developed into metastases at the experiment endpoint. This suggests that inhibition of IL-1ß signaling prevents colonization of DTCs rather than DTC homing to bone [58]. Consistently, pre-treatment with anakinra (1 mg/kg/day) has been shown to reduce the number of mice that developed bone metastases when compared to control (10% vs. 90%, respectively) [55]. The authors also report that inhibition of IL-1ß signaling reduces the number of CD-34^+^ micro-vessels in both, primary and bone metastatic MDA-MB-231-IV tumors in vivo using a preventative (treatment at day-3) and treatment schedule (treatment at day 7 post tumor cell injection) of anakinra (1 mg/kg/day) [55]. Indeed, IL-1ß has been shown to be required for tumor angiogenesis in vivo [107]. Moreover, humanized mouse models, where Anakinra (1 mg/kg/day) prevented breast cancer metastasis to human bone implants (0% vs. 57%) [108]. In naïve, tumor-free BALB/c mice a single dose of 1 mg/kg anakinra reduced osteoclast and osteoblast activity measured by serum TRAP and P1NP levels, respectively, as early as 4 h post injection [55]. This suggests that anakinra might also target bone cells by which it could reduce bone resorption and the availability of bone-derived, tumor-promoting factors. Consistently, formation of the bone metastasis niche and progression of osteolytic bone metastasis was recently shown to be regulated via the NAT1/NF-kB/IL-1ß axis [109].

#### 2.5.2. IL-6

Osteoblasts produce increased levels of inflammatory cytokines (e.g., IL-6 and IL-8) upon stimulation with breast cancer cells [96,98]. Vice versa, mesenchymal derived cells can induce STAT-3 signaling in breast cancer cell lines via IL-6, thereby enhancing breast cancer cell growth in vitro and in vivo [110]. Consistently, global gene expression analysis revealed that an IL-6 gene expression signature induced by the interaction of osteoblasts and breast cancer cells correlates with a shorter time to the development of bone metastasis in vivo [111]. Consequently, tumor cells might stimulate the production of osteoblast-derived cytokines including IL-6, which results in increased osteoclast differentiation and subsequent bone destruction. In line with these data, co-culture systems of osteoblasts and breast cancer cells report a decreased production of osteocalcin in addition to increased secretion of inflammatory cytokines including IL-6 [112].

Furthermore, bone marrow stromal cells produce IL-6 [28], which can induce osteolysis via crosstalk with the prostaglandin E_2_ (PGE_2_)/cyclooxygenase 2 (COX2) signaling cascade through altering the RANK/RANKL/OPG ratio [36]. Importantly, anti-IL-6 receptor treatment with tocilizumab suppresses RANK on MDA-MB-231 breast cancer cells, making them less susceptible to RANKL signaling [113]. In vivo, the anti-human IL-6 receptor monoclonal antibody reduced bone metastases derived from MDA-231 cells in mice when compared to control (93.8% of untreated mice vs. 60% of treated mice developed metastasis) [113]. Furthermore, knockdown of OSM, an IL-6 family cytokine, in 4T1 breast cancer cells (4T1.2-OSM) nearly abolished the development of spontaneous metastasis when 4T1 cells were injected orthotopically in BALB/c mice [88]. Mean metastatic burden in the spine was significantly reduced in mice injected with 4T1.2-OSM cells. After injection of those cells or control cells directly into the tibiae of mice, a decreased osteolysis was observed in the 4T1.2-OSM mice [88].

It has been proposed that senescent cells in the bone environment can impact metastasis initiation and progression via IL-signaling [114]. The effect of senescent osteoblasts on tumor cell recruitment and bone colonization was investigated using a specialized mouse model that allows the induction of senescence in mesenchymal cells. Indeed, senescent osteoblasts provided a supportive niche for DTCs, increased tumor cell seeding in the bone microenvironment as well as metastatic growth [114]. These effects were potentially caused through IL-6 from senescent osteoblasts that enhanced local osteoclastogenesis, rather than via direct effects of IL-6 on tumor cells. In agreement, inhibition of IL-6 with a neutralizing antibody reduced tumor cell growth in bone in this model [114]. Supporting these finding, others demonstrated that tumor-derived Jagged 1 activates Notch signaling and increases IL-6 in osteoblasts, promotes osteoclast differentiation and thereby supports breast cancer bone metastasis [115]. Separate studies investigating the osteoblast-breast cancer interaction during breast cancer bone metastasis showed that osteoblasts increase IL-6 in breast cancer cells via RANKL, resulting in a positive feedback loop [16]. Knockdown of RANK or IL-6 in cancer cells, or administration of an anti-IL-6 receptor antibody reduced tumor growth in bone [16].

In summary, most studies report a pro-tumorigenic role of IL-6. In contrast, some have analyzed the paracrine factors responsible for modulating osteoclast activity in breast cancer conditioned medium and found that conditioned media fractions could both, increase or inhibit osteoclasts [116]. The resorption-stimulating fraction contained TNF-α and insulin-like growth factor 2, whereas the inhibitory fraction contained granulocyte-macrophage colony-stimulating factor (GM-CSF), macrophage colony-stimulating factor (MCSF), IL-6 and TGF-β2 [116].

#### 2.5.3. IL-8

Several studies report a supportive role of both, autocrine and paracrine signaling of IL-8 in breast cancer bone metastasis [29,47]. Indeed, IL-8 can stimulate osteoclastogenesis, the hallmark of breast cancer bone metastasis, indirectly via increasing the RANKL on osteoblasts or directly via CXCR1 on osteoclasts [29]. Consistently, transgenic mice expressing human IL-8 demonstrated an osteopenic phenotype with elevated bone resorption [47]. Another striking finding in this mouse model was the increase in marrow adiposity as marrow adipocytes and associated adipokines are known to contribute to breast cancer bone metastasis [8,117]. In vitro studies have reported the secretion of two different isoforms of IL-8 by breast cancer cells, both being able to stimulate osteoclastogenesis [47]. Furthermore, the IL-8 expression in breast cancer cell lines correlated with their osteolytic potential in vitro and in vivo [118]. Additionally, mice treated with an anti-IL-8 antibody (35 µg/mouse) starting seven days after intratibial inoculation of breast cancer cells reduced tumor growth in bone when compared to control or untreated mice [47]. In a clinical setting, patients with breast cancer bone metastasis had increased levels of circulating IL-8, which correlated with elevated bone resorption markers when compared to patients without bone metastases [47]. In addition, osteoblast conditioned medium has been shown to increase IL-8 expression in human breast cancer cells [119], which could consequently stimulate osteoclastogenesis and the vicious cycle of bone metastasis. Consistently, others have shown that tumor cell-derived semaphorin 4D (Sema-4D) stimulated the production of IL-8 in osteoblasts to increase osteoclastogenesis in vitro [120]. In vivo inhibition of Sema4D in cancer cells by shRNA reduced metastatic breast cancer burden in mice when compared to mice injected with control cells [120].

Another microenvironmental component that is detrimental for tumor growth and progression includes vascular endothelial cells. Indeed, studies have shown that IL-8 stimulated chemotaxis and induced proliferation of human umbilical vein endothelial cells [121]. Moreover, hydron pellets containing IL-8 implanted into normally avascular rat cornea induced a corneal angiogenic response. In these studies the authors studied rheumatoid, inflammatory conditions and propose macrophages to be the source of IL-8 [121]. Studies have also shown that vascular endothelial cells in human breast cancer express receptors for IL-8, suggesting that IL-8 may also control proliferation, angiogenesis and metastasis in breast cancer via vascular endothelial cell activation [122]. In support, recombinant human IL-8 has been shown to induce neovascularization in a rabbit corneal pocket model [123].

#### 2.5.4. IL-11

Another prominent IL involved in breast cancer colonization and initiation of osteolytic disease in bone is IL-11. Bone marrow stromal cells have been shown to produce elevated levels of IL-11 in response to either tumor-derived factors or factors released during bone remodeling (e.g., TGF-β) [124]. This would then increase bone resorption, as IL-11 has been described to promote osteoclast differentiation and function [28,125]. Indeed, osteoclast differentiation induced by 1,25-dihydroxyvitamin D3, PTH, IL-1 or TNF-α could be blocked by neutralization of IL-11 [28]. Separate studies report that IL-11 promotes osteoclastic bone resorption via the prostaglandin E2 axis [125]. As cyclooxygenase inhibitors suppressed the production of prostaglandin E2 in calvaria cultures and consequently the IL-11 mediated bone resorption, cyclooxygenase inhibitors have been proposed as potent drugs to suppress IL-11 mediated osteolytic bone disease [125]. Others determined the role of breast cancer-derived IL-11 in osteoclastogenesis [126]. Both, medium conditioned by cancer cells and IL-11 supported the development and survival of osteoclast progenitor cells in vitro. By neutralizing IL-11 in the conditioned medium the authors showed that IL-11 was indeed the predominant cancer-derived factor that affects osteoclast precursor development and/or survival [126]. Recent studies have also shown that microRNA miR-124 reduces the survival of MDA-MB-231 breast cancer cells in the in vivo bone microenvironment and repressed cancer cell-induced osteolytic disease after intratibial injection via downregulation of IL-11 [127]. MiR-124 inhibited IL-11 and consequently osteoclastogenesis and bone metastasis. In vitro miR-124 inhibited osteoclastogenesis directly and indirectly by increasing RANKL/OPG in osteoblasts [127].

## 3. Other ILs in Breast Cancer Bone Metastasis

Besides IL-1, IL-6, IL-8 and IL-11, other ILs have been investigated in the context of breast cancer bone metastasis. IL-17A for example, has been shown to promote proliferation, migration and invasion of several breast cancer cell lines [128,129]. Studies have also linked autoimmune arthritis and breast cancer bone metastasis by an increased pro-inflammatory environment characterized by increased levels of circulating pro-inflammatory cytokines including IL-17 [130]. In mouse models of autoimmune arthritis 4T1 breast cancer lung and bone metastasis was increased when compared to non-arthritic mice [130]. Consequently, treatment with an anti-IL-17 antibody reduced breast cancer metastasis in two arthritic mouse models [130,131]. Studies have also associated IL-20 with breast cancer metastasis to bone [132]. IL-20 dose dependently increased breast cancer cell migration in vitro. Inhibition of IL-20 with a monoclonal antibody reduced mammary fad pad growth of 4T1 breast cancer cells and reduced breast cancer cell colonization in bone and osteolysis in vivo [132].

## 4. Conclusions and Summary

Patients with advanced breast cancer are at high risk of developing metastatic disease in the skeleton. Despite treatment advances, breast cancer bone metastasis remains incurable. A complex interaction between disseminated tumor cells and cells of the bone microenvironment drives disease progression in bone. The cancer-bone cell interaction is, at least in part, regulated by soluble factors including ILs. Several ILs have been shown to increase osteoclast activity, the hallmark of breast cancer-induced bone disease. Furthermore, in vitro studies have associated the expression of ILs with the metastatic capacity of breast cancer cell lines [20,37,51,52,53] and clinical studies report elevated serum IL levels in patients with bone metastasis [46,47]. The studies discussed in this review highlight the role of ILs in distinct events of the metastatic cascade—tumor cell dormancy, CSCs, migration and invasion as well as colonization of the metastatic site. Indeed, alterations in IL levels at the metastatic site could affect tumor cell homing to bone and determine whether the cells remain dormant or actively proliferate and proceed to form overt metastases. Thus, IL-based treatment strategies might be beneficial for patients who are at high risk of developing bone metastasis. Furthermore, targeting IL-signaling in bone cells could reduce the availability of tumor-growth promoting ILs in the bone microenvironment and thus slow down disease progression. In addition, the combination of agents that prevent IL-signaling with standard of care for cancer-induced bone disease might be favorable in patients with advanced breast cancer and/or for patients who experience treatment resistance.

Together, promising pre-clinical results support the potential of ILs as therapeutic targets for patients with advanced breast cancer and highlight the need to increase the understanding of the IL-mediated cancer-bone cell crosstalk in order to improve treatment outcome for patients with breast cancer bone metastasis.

## Figures and Tables

**Table 1:** Known interleukins grouped according to their respective receptor families. References for Table 1 [21,22,23].

Receptor Family	Interleukins
Type I cytokine receptor family	IL-12 family: IL-12, IL-23, IL-27, IL-30, IL-35 Glycoprotein gp130/IL-6 family: IL-6, IL-11, IL-31 β chain cytokine family: IL-3, IL-5 γ chain cytokine family: IL-2, IL-4, IL-7, IL-9, IL-13, IL-15, IL-21 others: IL-16, IL-32, IL-34
Type II cytokine receptor family	IL-10 family: IL-10 IL-20 subfamily: IL-19, IL-20, IL-22, IL-24, IL-26 Type III interferons (IFNs): IL-28A, IL-28B, IL-29
IL-1/Toll like receptor	IL-1 family: IL-1α, IL-1β, IL-1Ra, IL-18, IL-33, IL-36α, IL-36β, IL-36γ, IL-36Ra, IL-37, IL-38
Others	IL-14 IL-17 family: IL-17A-F, IL-25 (IL-17E) CXC chemokine family: IL-8
